# An Ecosystem-Service Approach to Evaluate the Role of Non-Native Species in Urbanized Wetlands

**DOI:** 10.3390/ijerph120403926

**Published:** 2015-04-09

**Authors:** Rita S. W. Yam, Ko-Pu Huang, Hwey-Lian Hsieh, Hsing-Juh Lin, Shou-Chung Huang

**Affiliations:** 1 Department of Bioenvironmental Systems Engineering, National Taiwan University, Taipei 106, Taiwan; E-Mail: d00622007@ntu.edu.tw; 2 Biodiversity Research Center, Academia Sinica, Taipei 115, Taiwan; E-Mail: zohl@gate.sinica.edu.tw; 3 Department of Life Sciences and Research Center for Global Change Biology, National Chung Hsing University, Taichung 402, Taiwan; E-Mail: hjlin@dragon.nchu.edu.tw; 4 Taiwan Wetland Society, Hsinchu City 300, Taiwan; E-Mail: huang120@gate.sinica.edu.tw

**Keywords:** urban ecosystems, alien species, exotic species, management, ecosystem services, Asian wetlands

## Abstract

Natural wetlands have been increasingly transformed into urbanized ecosystems commonly colonized by stress-tolerant non-native species. Although non-native species present numerous threats to natural ecosystems, some could provide important benefits to urbanized ecosystems. This study investigated the extent of colonization by non-native fish and bird species of three urbanized wetlands in subtropical Taiwan. Using literature data the role of each non-native species in the urbanized wetland was evaluated by their effect (benefits/damages) on ecosystem services (ES) based on their ecological traits. Our sites were seriously colonized by non-native fishes (39%–100%), but <3% by non-native birds. Although most non-native species could damage ES regulation (disease control and wastewater purification), some could be beneficial to the urbanized wetland ES. Our results indicated the importance of non-native fishes in supporting ES by serving as food source to fish-eating waterbirds (native, and migratory species) due to their high abundance, particularly for *Oreochromis* spp. However, all non-native birds are regarded as “harmful” species causing important ecosystem disservices, and thus eradication of these bird-invaders from urban wetlands would be needed. This simple framework for role evaluation of non-native species represents a holistic and transferable approach to facilitate decision making on management priority of non-native species in urbanized wetlands.

## 1. Introduction

In recent decades global urbanization has caused severe impacts on wetland ecosystems, including non-native species invasion, habitat deterioration and hydrological alteration [1,2,3]. Urbanized wetlands are unique and novel ecosystems, but commonly found worldwide [4,5]. They generally include degraded natural wetlands, wetlands constructed for various purposes (e.g., wastewater treatment, stormwater retention, recreation), channelized urban rivers, heavily-modified estuaries and coastal areas, aquaculture ponds, paddy fields and water ponds. Urbanized wetlands are characterized by profound human-induced changes in abiotic environments, and significant shifts in local biodiversity due to displacement of stress-sensitive native (or restored) species by competitive non-native species. As the landscape sinks, this could further enhance their susceptibility to non-native species invasion and reinvasion from the urbanized catchment via the inflowing floods and surface runoff [6]. Accumulated studies e.g., [2,7] have indicated that non-native species have become the dominant inhabitants in most urbanized wetlands. Various management efforts have been implemented for the removal of non-native species in these urbanized habitats [8] due to the traditional perception of their potential ecological and economic impacts [9,10], yet complete eradication of non-native species from urbanized wetlands has largely remained unsuccessful [4,5].

Undoubtedly, the ecosystem structure and processes in urbanized wetlands differ intrinsically from those of natural wetlands, and thus different management strategies are needed for these two types of ecosystems. However, the evaluation of non-native species tends to only focus on their negative effects because current ecological character assessments of wetlands are designed for natural wetlands [11]. In order to achieve the wise use of urbanized wetlands, the roles of non-native species in the urbanized wetlands must be carefully evaluated from a different angle instead of using the same assessment methods as for natural wetlands [12]. Recent studies have argued that a proportion of non-native species not only were relatively innocuous, but also could benefit the heavily impaired or novel ecosystems by providing important ecological benefits including food and habitat provision for native species, catalyzing ecosystem restoration by enhancing the structural complexity of habitats and species richness, augmenting ecosystem services (ES) [13,14]. These benefits to urbanized wetlands are, however, underreported. Therefore, this could have commonly caused the mismanagement of the beneficial non-native species in urbanized wetlands.

The ES approach has been increasingly advocated to facilitate decision making associated with non-native species assessment and management [9,15]*.* According to the Millennium Ecosystem Assessment, ES assessment is an integrated method of measurement of the material and non-material importance of all structural and functional components of the ecosystems because they represent all the goods and benefits that people could derive from ecosystems directly or indirectly, including provisioning, regulation, cultural and supporting services [16]. Thus, linking the non-native species effects to the ES could be one of the best approaches to understand their roles in urbanized wetlands. However, the scientific knowledge for identifying the role of each non-native species as ecosystem service or disservice providers in urban ecosystems is generally limited, particularly for Asia [12,17,18].

Subtropical wetlands are characterized by hydrological extremes resulted from strong seasonality in precipitation and intensive human impacts. However, these wetlands are used to support high biodiversity [19,20] and represent important breeding, wintering and stop-over sites for migratory birds [21]. As the consequence of extensive urbanization in lowland and coastal areas, natural wetlands have been increasingly transformed into novel urbanized ecosystems dominated by non-native species. In spite of the difficulty of complete eradiation of non-native species in such environments, it would be important to prioritize the management effort on the most “harmful” non-native species, which cause only damages on ES, so as to maximize ES and minimize disservices. Hence, there is pressing need for establishing a simple and integrated approach to enable wetland managers to identify the role of different non-native species on urbanized wetland ES with respect to their ecological traits.

In this study, we aimed to determine the non-native species that could have beneficial roles on the urbanized wetland ES. We first investigated the colonization extent of the non-native fish and bird species of three urbanized wetlands located in subtropical northern Taiwan. A qualitative assessment was carried out to evaluate the role of each non-native species in urbanized wetlands by the number of their different effect types (benefits/damages) on ES based on their ecological traits using literature data. Finally, a framework for evaluating the role of non-native species in urbanized wetlands based on ES approach was suggested. Results should be helpful to facilitate the decision making for the management priority of different non-native species so that eradication efforts could be directed to those “harmful” non-native species with only negative impacts on urbanized wetland ES. Other non-native species able to tolerate severe human-disturbances and exert beneficial effect(s) on the ES could be temporarily retained to facilitate ecological restoration of the urbanized ecosystems.

## 2. Methods and Materials

### 2.1. Study Sites

**Figure 1:**
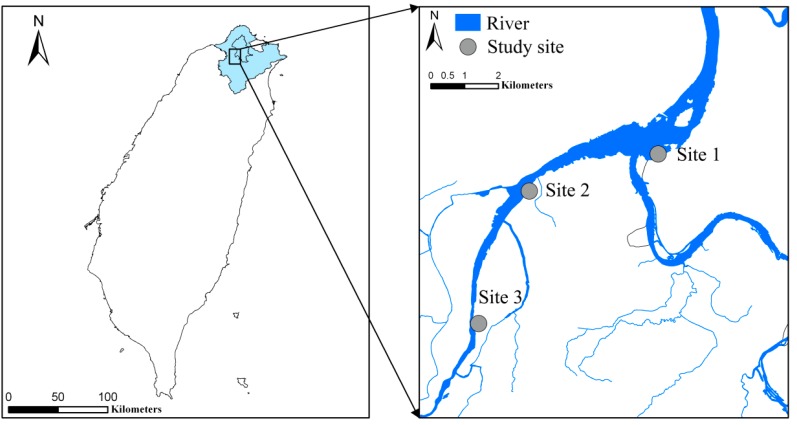
Map showing the locations of the three study urbanized wetlands. Site 1 = Huajiang Wetland; Site 2 = Hsin-Hai Constructed Wetland; Site 3 = Daniaopi Constructed Wetland.

Taiwan is a typical monsoon island in East Asia and located along the East Asian-Australasian flyways for migratory birds [21]. The present study was conducted in Huajiang Wetland (Site 1: 25°02' N, 121°29' E), Hsin-Hai Constructed Wetland Phase 2 (Site 2: 25°01'N, 121°27'E), and Daniaopi Constructed Wetland (Site 3: 24°59' N, 121°26' E) and from the highly urbanized region in the subtropical northern Taiwan (Figure 1). This region is influenced by a subtropical climate with wet summers and mild winters (annual mean temperature: summer = 29.6 °C; winter = 16.1 °C) with high annual precipitation (annual mean rainfall = 2405.1 mm) [22]. All three study sites are permanent wetlands along the floodplain of the highly urbanized Tamsui River basin, they are influenced by the same climate, catchment landscape and land-use pattern [23]. Site 1 is a degraded natural wetland in an urbanized landscape. It has been part of the Taipei City Waterbird Refuge for habitat protection and conservation of migratory birds since 1997. Sites 2 and 3 are free-water-surface constructed wetlands completed in 2006 [24]. These two sites were designed to serve for multiple functions such as wastewater treatment for domestic sewage from surrounding urbanized areas, habitat provision for wildlife, and environmental education. The total area of Site 1 (82.7 ha) is larger than Site 2 (4.7 ha) and Site 3 (13.0 ha) (Table 1). Elevated nutrient levels were measured at these three sites (mean NH_4_-N = 3.39–9.66 mg/L; mean NO_3_-N = 0.44–3.21 mg/L; mean TP = 0.77–3.99 mg/L). Hypoxic conditions commonly occurred at all sites (Table 1) despite the water temperature being <23 °C throughout our study period.

**Table 1:** Environmental characteristics of the three study urbanized wetlands including wetland type, total area, wetted area, and mean ± SE of nutrient and dissolved oxygen concentrations.

Site	Type	Total Area (ha)	Wetted Area (ha)	NO_3_-N (mg/L)	NH_4_-N (mg/L)	TP (mg/L)	DO (mg/L)
1	DNW	82.7	20.7	3.21 ± 0.55	5.78 ± 0.74	0.77 ± 0.12	2.37 ± 0.43
2	FWS	4.7	3.3	0.44 ± 0.13	9.66 ± 1.41	3.99 ± 1.19	3.44 ± 0.67
3	FWS	13.0	8.5	0.49 ± 0.08	3.39 ± 0.71	1.26 ± 0.40	6.41 ± 1.05

DNW = degraded natural wetland; FWS = free water surface constructed wetland. NO_3_-N = nitrate-nitrogen, NH_4_-N = ammonium-nitrogen, and TP = total phosphorus; DO = dissolved oxygen. See Figure 1 for site codes.

### 2.2. Collection of Biodiversity Data

Basically, fish and bird biodiversity data of Site 1 was extracted from [25], and that of Sites 2 and 3 were extracted from [20]. Additional fish sampling was conducted twice in Site 1 during October 2008 to February 2009, and in Sites 2 and 3 during October 2009 to February 2010 by the present study to collect any additional species not recorded in [20,25]. Both cast net and shrimp traps were used for fish sampling at all study sites. The relative importance of non-native species of the fish community calculated was (1) the relative species richness (number of non-native species/total number of fish species) and (2) the relative abundance (abundance of non-native species/total abundance of all fish species) for comparing the fish community structure among our study wetlands to avoid any potential sampling bias. The fish abundance was calculated as number of fish catch per unit sampling effort hereafter. Bird observation was conducted by trained persons three times in Site 1 during October 2008 to February 2009, and in Sites 2 and 3 during October 2009 to February 2010. The bird abundance of each site was defined as number of observation per unit sampling effort hereafter. Non-native fish and bird species of wetlands from Taiwan were identified based on the definition that species occurring in these sites were outside their natural range and dispersal potential (with or without direct and indirect human-mediated introduction) [26,27,28]. Also, the dispersal characteristics (*i.e.*, migratory or non-migratory) and habitat preference (*i.e.*, waterbirds or landbirds) of bird species from our study sites were determined based on [28,29].

### 2.3. Assessing the Effects of Non-Native Species on the ecosystem services (ES)

Quantification of ES in urbanized wetlands can be complex due to the lack of commonly recognized monetary values of different ecological functions and dysfunctions in such ecosystems associated with different human groups with various interests. Therefore, only number of different effect types of the non-native species on the ES of urbanized wetlands was assessed in this study. The ES provided by our three study wetlands were determined based on the three ES categories, including regulating, cultural and supporting services, from the Millennium Ecosystem Assessment [16]. But, food, energy and raw material provisioning services to humans were not considered due to their negligible local importance. The role of each non-native species from the study urbanized wetlands was evaluated as the ES or disservice provider based on their effect types (benefit or damage) on ES by examining their ecological trait data from international and local ecological studies.

## 3. Results

### 3.1. Colonization Extents of Non-Native Species in Urbanized Wetlands

**Figure 2:**
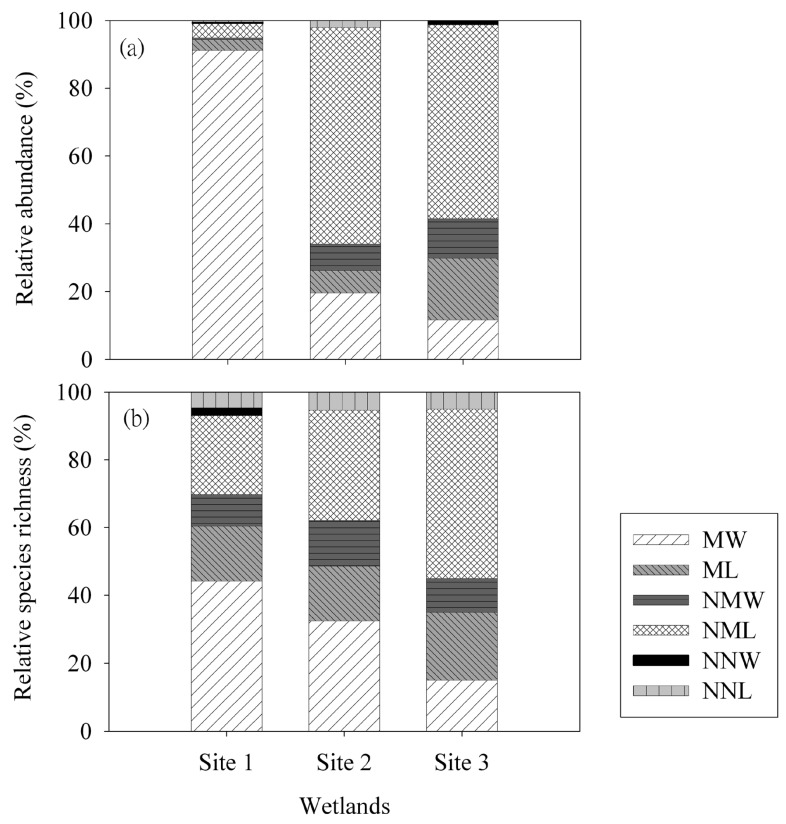
The percentages of (**a**) the relative abundance (observation per unit sampling effort) and (**b**) the relative species richness of birds for different ecological categories recorded in the three study wetlands. Different ecological categories of birds: MW = migratory waterbirds; ML = migratory landbirds; NMW = non-migratory waterbirds; NML = non-migratory landbirds; NNW = non-native waterbirds; NNL = non-native landbirds. See Figure 1 for site codes.

Despite the low species richness of fish was recorded at all sites (Site 1 = 6 species; Site 2 = 2 species; Site 3 = 4 species), their fish communities were dominated by the non-native species with high abundance (39%–100%; Table 2), but, the relative abundance of non-native fish from Sites 2 and 3 were almost twice as high as that from Site 1. Three-spot gourami (*Trichogaster trichopterus*) and tilapia (*Oreochromis* spp.) represented the two most dominant non-native fish species in our sites (Table 3). In particular, the highest relative abundance of *Oreochromis* spp. were recorded in both Site 1 (32.26%) and Site 3 (72.61%). The three studied urbanized wetlands showed inconsistent bird species richness (Site 1 = 27 species; Site 2 = 20 species; Site 3 = 37 species) (Table 2). In contrast to the enormous extent of non-native fish colonization in our study wetlands, the relative abundances of non-native birds at all three sites were low (<3%). Most birds recorded in Site 1 were winter migratory waterbirds comprising of 86.16% of the total bird abundance whilst Sites 2 and 3 were dominated by non-migratory landbirds (Site 2 = 57.14% and Site 3 = 63.87%; Figure 2a,b). Four non-native bird species were commonly observed in our three wetland sites, including one non-native waterbird species (sacred ibis *Threskiornis aethiopicus*) and three non-native landbird species (rock pigeon *Columba livia*, white-vented myna *Acridotheres javanicus*, and common myna *Acridotheres tristis*) (Table 3).

**Table 2:** Relative species richness and abundance of non-native fish and bird species in our three study urbanized wetlands.

Site	Fish				Birds			
	Species Richness		Abundance		Species Richness		Abundance	
	Non-Native Species/All Species	%	Non-Native Species/All Species	%	Non-Native Species/All Species	%	Non-Native Species/All Species	%
1	3/6	50	6.00/15.50	38.71	3/27	11.11	8.67/385.33	2.25
2	2/2	100	120.05/120.05	100	1/20	5.00	0.33/25.67	1.30
3	1/4	25	57.00/78.50	72.61	2/37	5.41	2.67/131.00	2.04

Unit of fish abundance = catch per unit sampling effort; units of bird abundance = observation per unit sampling effort. See Figure 1 for site codes.

**Table 3:** Abundance of the dominant non-native fish and bird species in the three study urbanized wetlands.

Non-Native Species	Site 1	Site 2	Site 3
Fishes			
*Carassius cuvieri*	0.50	0.00	0.00
*Channa striata*	0.50	0.00	0.00
*Oreochromis spp.*	5.00	19.50	57.00
*Trichogaster trichopterus*	0.00	101.00	0.00
Birds			
*Acridotheres javanicus*	0.00	0.33	2.00
*Acridotheres tristis*	0.33	0.00	0.00
*Columba livia*	5.67	0.00	0.67
*Threskiornis aethiopicus*	2.67	0.00	0.00

Unit of fish abundance = catch per unit sampling effort; units of bird abundance = observation per unit sampling effort. See Figure 1 for site codes.

### 3.2. Effects of Non-Native Species on Urbanized Wetland ecosystem services (ES)

#### 3.2.1. Regulating Services

According to the results of literature mining of the ecological traits of the non-native fish and bird species, none of them have any beneficial role in regulating any ES in the study urbanized wetlands. Most (including *Channa striata*, *Oreochromis* spp., *Trichogaster* *trichopterus*, *Acridotheres* *tristis*, *Columba* *livia*, *Threskiornis* *aethiopicus*) were vectors of diseases and they could cause potential health hazards to the native fauna and even human beings (Table 4). Also, the extensive sediment bioturbation effect caused by abundant *Oreochromis* spp. via bottom feeding and spawning activities could enhance the release of nutrients (phosphate and ammonia) from sediments into the overlying water, thus increasing aqueous nitrogen (N) and phosphorus (P) availability [30,31,32,33]. Therefore, the presence of *Oreochromis* spp. could highly exacerbate the eutrophic condition, and impact the ES of wastewater purification of the urbanized wetlands.

**Table 4:** Effect types (damage = “−”; benefit = “+”) of non-native fish and bird species on the ecosystem services of our study urbanized wetlands. Effect types were classified into three categories including supporting, regulating and cultural services.

Non-Native Species	Ecosystem Services	Degraded Natural Wetland			Free-Water-Surface Constructed Wetlands		
		Effect Types		References	Effect Types		References
**Fish**							
*Carassius cuvieri*	**Supporting services**	−+	**Biodiversity:** Competing with native fishes for food and space; Serving as minor food sources for birds	[27,34]			
*Channa striata*	**Regulating services**	−	**Disease regulation:** Vectors of diseases	[35]			
	**Supporting services**	−	**Biodiversity:** Highly predacious and threatening native fauna through aggressive predation and competition	[27,35]			
*Oreochromis spp.*	**Regulating services**	−	**Wastewater purification:** Release of N and P from sediment through bioturbation	[30,31,32,33]	−	**Wastewater purification:** Release of N and P from sediment through bioturbation	[30,31,32,33]
		−	**Disease regulation:** Vectors of diseases	[32,36]	−	**Disease regulation:** Vectors of diseases	[32,36]
	**Supporting services**	− +	**Biodiversity:** Displacing native fishes through competition;Providing major food sources for birds	[27,29,37,38,39,40,41]	− +	**Biodiversity:** Occupying ecological niches of native species and hindering their colonization; Providing major food sources for birds	[27,29,37,38]
		−	**Nutrient cycling:** Alteration of nutrient cycling through increasing aqueous N and P availability	[30,31,32,33,40]	−	**Nutrient cycling:** Alteration of nutrient cycling through increasing aqueous N and P availability	[30,31,32,33,40]
*Trichogaster trichopterus*	**Regulating services**				−	**Disease regulation:** Vectors of diseases	[42]
	**Supporting services**				−	**Biodiversity:** Occupying ecological niches of native species and hindering their colonization;	[27]
Birds							
*Acridotheres javanicus*	**Supporting services**				−	**Biodiversity:** Food and nest-site competitors with native birds	[10,43]
	**Cultural services**				+	**Recreational service:** Visitors gaining psychological well-being via exposure to birds in stressful urbanized environment	[44,45]
*Acridotheres tristis*	**Regulating services**	−	**Disease regulation:** Vectors of diseases	[46]			
	**Supporting services**	+ −	**Biodiversity:** Food and nest-site competitors with native birds, e.g., the endemic Crested Myna (*Acridotheres cristatellus*) Pollination of native plants	[29,43,46,47]			
*Columba livia*	**Regulating services**	−	**Disease regulation:** Vectors of diseases	[43,47,48]	−	**Disease regulation:** Vectors of diseases	[43,47,48]
	**Cultural services**				+	**Recreational:** Visitors gaining psychological well-being via exposure to birds in stressful urbanized environment	[44,45]
	**Supporting services**	−	**Biodiversity:** Competing with native landbirds	[10,47]	−	**Biodiversity:** Occupying ecological niches of native species and hindering their colonization;	[10]
*Threskiornis aethiopicus*	**Regulating services**	−	**Disease regulation:** Vectors of diseases	[49]			
	**Supporting services**	−	**Biodiversity:** Competing with native Ardeidae species for habitat and foraging area	[50]			
	**Cultural services**	+	**Recreational:** Visitors gaining psychological well-being when seeing large waterbirds in stressful urbanized environment	[44,45,51]			

#### 3.2.2. Supporting Services

Non-native fish species exerted various effects on supporting services. Through a number of negative interactions including predation, competition, niche occupancy of native species, all four dominant non-native fishes caused damages on the supporting services of our study urbanized wetlands (Table 4). This resulted in the observed low species richness and abundance of native fish species of our study urbanized wetlands (Table 2). However, due to the consistently high abundance of the non-native fish, these species could serve as important food resource for predatory waterbirds, e.g., Ardeidae and Scolopacidae. All non-native bird species, *i.e.*, *Acridotheres* *javanicus*, *Acridotheres* *tristis*, *Columba* *livia*, *Threskiornis* *aethiopicus* could directly damage the supporting ES of urbanized wetlands because of their strong competitive ability and aggressiveness. These characteristics thus enable them to act as potential resource-competitors of native birds and migratory species.

#### 3.2.3. Cultural Services

The presence of birds, even for non-native species, in the urbanized ecosystems could benefit the cultural ES by enhancing the recreational potential for the general public and visitors to gain psychological well-being via exposure to birds in the stressful urbanized environment [44,45]. In particular for the two constructed wetland sites, *i.e.*, Sites 2 and 3, the presence of non-native birds *Acridotheres javanicus* and *Columba livia* could benefit the recreational services by enriching local bird biodiversity of the urbanized wetland sites (Table 4).

## 4. Discussion

### 4.1. Biodiversity of Urban Wetlands

Our study wetlands were typical urbanized riverine wetlands with serious habitat deterioration due to the intensive water pollution and hypoxia associated with the wastewater discharges from the urbanized catchments of the Taipei metropolitan region [52]. Also, all study sites were predominantly colonized by non-native fishes (Table 2). Since historical pollution and human-caused/mediated fish invasions of the urbanized Tamsui River and Dahan River, fish communities from these rivers have long been dominated by the pollution-tolerant non-native fishes (>80%) [53,54]. Moreover, the few competitively superior species as characterized by rapid growth, high fecundity, strong territoriality (e.g., *Oreochromis* spp.), long-term parental care (e.g., *Trichogaster* *trichopterus*), and/or aggressive predatory ability (e.g., *Channa striata*), further enhanced their dominance in the urbanized rivers, and thus successful invasion and colonization in all study wetland sites on the associated floodplains [27,54]. However, the colonization of non-native fish species (in term of species richness) was not consistent among the three study sites (Table 2 and Table 3) despite their high similarity in physical environmental properties such as geographical location, climatic influence and similar flood-disturbance regime. Our findings supported the contention of previous studies that biological invasion to urban wetlands could be site-specific as non-native species could have individualistic responses to the integrated influence of various environmental properties, biotic interactions and anthropogenic effects in urban wetlands [2,8]. In addition, the colonization by non-native fishes (in terms of relative abundance) in Sites 2 and 3 were ≥2 times higher than Site 1. As free-water-surface constructed wetlands, Sites 2 and 3 received large amount of urban sewage due to their primary function as wastewater treatment areas. This has therefore created a niche for the tolerant and competitive non-native fishes which could adapt well to the continuous pollution stress in urbanized wetlands, e.g., [20,27,40].

In contrast to the high extent of colonization by non-native fishes, non-native birds comprised only <3% of species in our study urbanized wetlands. Bird species recorded in our study wetlands were mainly native and migratory (Figure 2), this confirmed the importance of our study urbanized wetlands as their habitats in the urbanized landscape. Also, our findings were consistent with other ecological studies on constructed wetlands indicating that poor water quality features of wetlands such as eutrophication and hypoxia, had no significant detrimental impacts on bird distribution and abundance, e.g., [5,55]. Instead, large habitat size, low human disturbance intensity, and diverse coverage ratios of wetted area/bare ground (for habitat requirements of different species) were considered as key determinants for good wetland quality for waterbirds [56]. This therefore explained the highest species richness and abundance of migratory waterbirds recorded in Site 1 as compared Sites 2 and 3. Despite the relative low species richness and abundance of non-native birds from all sites, their potential damages and benefits must be properly evaluated in urbanized wetland ecosystems as these bird species were the common invaders in most urbanized wetlands worldwide [28,46,57].

### 4.2. Benefits and Damages of Non-Native Species to the ecosystem services (ES) of Urbanized Wetlands

Most non-native fish and bird species have similar role for causing ecosystem disservices in the urbanized wetlands from subtropical Taiwan including threatening native biodiversity via competition, being vectors of diseases and hindering wastewater purification of the urbanized wetlands (Table 4). Such results were consistent with several previous studies for other parts of the world [3,7]. Nonetheless, our results highlighted that some non-native species could be beneficial to the urbanized wetland systems. From the perspective of the general public, the presence of non-native birds, such as *Acridotheres* *javanicus* and *Columba* *livia*, could enrich the local bird species richness and thus benefit the cultural ES through increasing the recreational values for visitors of the constructed wetlands particularly for the newly established sites with relatively low biodiversity and abundance of migratory birds [24,44,45,56]. In addition, the highly abundant non-native fishes, particularly for *Oreochromis* spp., in our study urbanized wetlands could potentially provide important trophic support to the waterbirds including migratory species from the East Asian–Australasian flyways [21].

Accumulated studies have confirmed that the diets of fish-eating waterbirds could shift from native fish to non-native fish following the introduction of non-native fish to wetlands [58,34]. Local ecological studies of waterbirds in Taiwan confirmed that abundant non-native fishes such as tilapia *Oreochromis* spp. could contributed ~20% to the diets of fish-eating native and migratory birds, e.g., Black-winged Stilt (*Himantopus himantopus*), and Black-faced Spoonbill (*Platalea minor*) [37,38]. Non-native fishes could also serve as major prey for the fish-eating native and migratory birds, e.g., Ardeidae and Scolopacidae [29]. Other studies confirmed that non-native tilapia was the most important food resources to support the populations of fish-eating birds in urban inland lakes and coastal wetlands due to their high abundance, e.g., [59].

Based on the effect types of different non-native species on the urbanized wetland ES, all non-native birds are regarded as “harmful” non-native species by causing more than one important ecosystem disservice for the supporting and regulating aspects and their effects could potentially cause the profound environmental degradation and ecosystem changes [9,10]. We suggested that eradication would be needed for these international common invaders (e.g., *Acridotheres* *javanicus*, *Acridotheres* *tristis*, and *Columba* *livia*) [57,46] although they had one general beneficial effect for cultural services of the urbanized wetlands (Table 4), and their current colonization extent was limited in our sites (Figure 2).

For the non-native fish, *Oreochromis* spp. has the highest number (>2) of negative impact types on supporting and regulating services of urbanized wetland ES whereas *Channa* *striata*, *Trichogaster* *trichopterus* and *Carassius cuvieri* have 1–2 negative impact types. However, as some non-native fish (*Oreochromis* spp. and *Carassius* *cuvieri*) are potential food resources for the ecologically important native and migratory waterbirds, they could serve as substitutes for native fish prey to provide trophic support for these birds in the highly polluted urbanized wetlands [14,15]. *Oreochromis* spp. in our sites was therefore not considered as a “harmful” non-native species in the urbanized wetlands and immediate eradication would not be suggested for this species. Also, we did not suggest for eradicating the other three dominant non-native fishes, *i.e.*, *Channa* *striata*, *Trichogaster* *trichopterus* and *Carassius cuvieri*, from our sites because of their high tolerance to the pollution stress in the urbanized wetlands. In the highly disturbed environment of urbanized wetland ecosystems, it would be a futile effort for removing the tolerant non-native species before complete eliminating or at least significantly reducing the disturbances or their natural and human drivers, e.g., repeatedly invasion of non-native fishes from the adjoined urbanized rivers associated with floods, input of pollutants and excess nutrients from the adjoined urbanized catchments. Hence, the wise management of urbanized wetlands should consider other options instead of complete eradication of the non-native species, *i.e.*, identification and making use of the non-native species able to tolerate severe human-disturbance and exert positive effect(s) on the ES to facilitate ecological restoration. Eradication effort could then be directed to those “harmful” non-native species with only negative impacts on wetland ES for effective allocation financial and labour resources.

### 4.3. Framework for Evaluating the Role of Non-Native Species in Urbanized Wetlands Based on ecosystem services (ES) Approach

**Figure 3:**
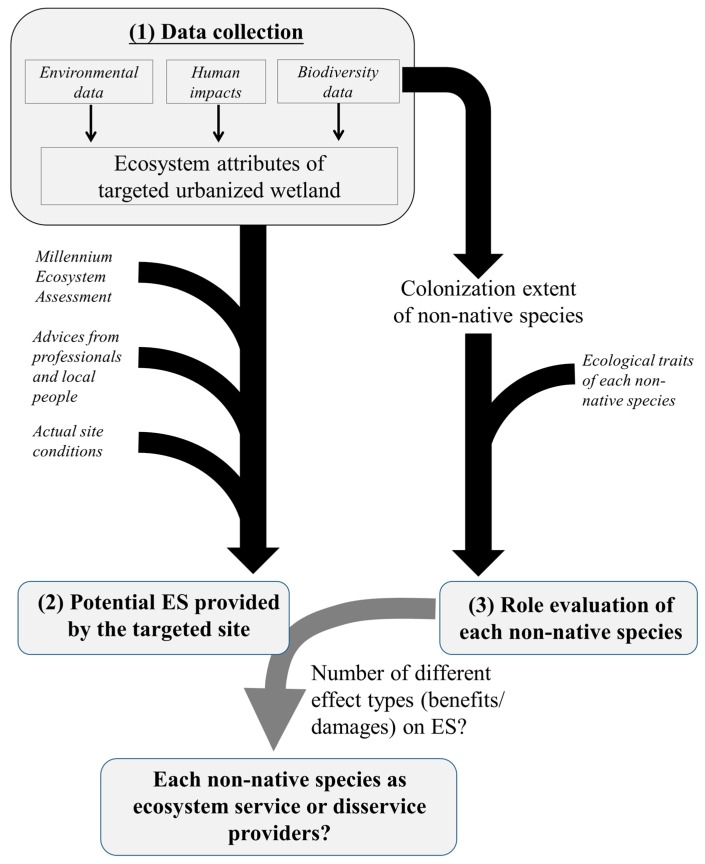
Conceptual framework for evaluating the role of non-native species in urbanized wetlands based on ES approach.

The role evaluation of each non-native species in urbanized wetlands based on the qualitative (*i.e.*, non-monetary) assessment of the overall effects (benefits/damages) on ES of the targeted sites with respect to the number their various effect types should involve three major components including (1) environmental and biodiversity data collection; (2) estimation of the potential ES of targeted sites; (3) qualitative assessment to evaluate the role of each non-native species by the number of their different effect types (benefits/damages) on ES based on their ecological traits using literature data (Figure 3). Data collection represents an important step to develop the basic understanding of not only climatic, geographical, hydrological and ecological conditions, but also the extents of human impacts and non-native species colonization of the targeted urbanized wetland. Environmental and biodiversity data could be retrieved from historical databases (if existent) and/or collected by periodic field surveys.

The potential ES of the targeted urbanized wetland would be determined by examining the literature elaborations of these environmental and biodiversity attributes including climate, geography, hydrology and ecosystem structure, as well as the combination of these attributes with natural and cultural attractions (e.g., sight-seeing landmarks, attractive species). The potential ES provided by the targeted urbanized wetlands could be identified based on the Millennium Ecosystem Assessment [16]. Specific types or categories of potential ES would be added or deleted according to the actual site conditions and/or professional advices from scientists and local people (particularly when the relevant literature is lacking). Finally, the role evaluation of each non-native species in the targeted urbanized wetland would be carried out by determining the number of their different effect types (benefits/damages) on ES based on the compiling literature information to determine their ecological traits of non-native species from international and local studies.

This assessment represents a simple and transferable approach to integrate the effects of the non-native species on ES and determine each species as ecosystem service or disservice providers in urbanized wetlands [17,60]. Results could be helpful for wetland managers to prioritize the efforts to eradicate and control “harmful” non-native species only to those causing (serious) negative effects on the important ES of urbanized wetlands. The colonization extent and ecological role(s) of non-native species need to be evaluated regularly in order to update our understandings on the effects of non-native species and modify the management direction in the rapid changing/evolving urbanized wetland ecosystems.

## 5. Conclusions

Our findings indicated that most non-native species could damage ES regulation (disease control and wastewater purification), but some could be beneficial to the urbanized wetland ES. Based on the effect types of different non-native species on the urbanized wetland ES, all non-native birds are regarded as “harmful” species by causing >1 important ecosystem disservice to the support and regulation aspects and their effects could potentially cause profound environmental degradation and ecosystem changes. Thus, eradication of these international common bird-invaders (e.g., *Acridotheres* *javanicus*, *Acridotheres* *tristis*, and *Columba* *livia*) in our sites is suggested, despite their limited colonization extent and benefits to cultural service in urbanized wetlands. In contrast, some non-native fish (*Oreochromis* spp. and *Carassius* *cuvieri*) are potential food resources for the ecologically important native and migratory waterbirds, and they could serve as substitutes for native fish prey to provide trophic support for these birds in the highly polluted urbanized wetlands. As these species tolerate severe human-disturbances and exert beneficial effect(s) on the ES, so they could be temporarily retained to facilitate ecological restoration of the urbanized wetland ecosystems.

The framework presented in this study for evaluating the role of non-native species in urbanized wetlands based on an ecosystem services (ES) approach represents a holistic and transferable approach to integrate the various effects of the non-native species on ES and determine each species as an ecosystem service or disservice provider in urbanized wetlands. It could therefore facilitate decision making for the priority management of non-native species in urbanized wetlands.
